# PHYSICAL ACTIVITY, SCREEN TIME, NUTRITIONAL STATUS AND SLEEP IN ADOLESCENTS IN NORTHEAST BRAZIL

**DOI:** 10.1590/1984-0462/2021/39/2019138

**Published:** 2020-08-26

**Authors:** João Miguel de Souza, Filipe Ferreira da Costa, Arthur Oliveira Barbosa, Alcides Prazeres, Elaine Valdna Oliveira dos Santos, José Cazuza de Farias

**Affiliations:** a Universidade de Pernambuco, Recife, PE, Brazil.; b Universidade Federal da Paraíba, João Pessoa, PB, Brazil.

**Keywords:** Adolescent, Motor activity, Sedentary behavior, Nutritional status, Sleep, Adolescente, Atividade motora, Estilo de vida sedentário, Estado nutricional, Sono

## Abstract

**Objective:**

To verify the prevalence of sleep quality and duration and its association with the level of physical activity, screen time and nutritional status in adolescents.

**Methods:**

This is a cross-sectional study with 1,432 adolescents (53.1%, female), ten to 14 years old (12.0±1.0 year) from public schools in Joao Pessoa, Northeast Brazil. Physical activity (≥300 vs. <300 minutes/week), screen time (≤2 vs. >2 h/day) and duration (<8 vs. ≥8 h/day) and perception of sleep quality (negative vs. positive perception) were measured by questionnaire. Nutritional status was assessed by body mass index (low weight / normal weight vs. overweight / obesity). Binary logistic regression was used to analyze association between variables, considering different aggregation of negative factors.

**Results:**

The prevalence of insufficient duration and negative perception of sleep quality was 12.6% (95%CI 10.9-14.4) and 21.0% (95%CI 18.9-23.1), respectively. There was a linear trend in the chance of the adolescents to present insufficient duration of sleep as a result of simultaneous negative factors (physical inactivity, excessive screen time, being overweight) (OR=4.31; 95%CI 1.50-12.48).

**Conclusions:**

Adolescents exposed simultaneously to low levels of physical activity, excessive screen time and overweight had a lower sleep duration.

## RESUMO

**Objetivo:**

Verificar a prevalência da percepção da qualidade e duração do sono e sua associação com o nível de atividade física, tempo de tela e estado nutricional em adolescentes.

**Métodos:**

Trata-se de um estudo transversal com 1.432 adolescentes (53,1% do sexo feminino), de dez a 14 anos de idade (12,0±1,0 ano) e de escolas públicas de João Pessoa (PB). A atividade física (≥300 *vs.* <300 min/semana), o tempo de tela (≤2 *vs.* >2 h/dia) e a duração (<8 *vs.* ≥8 h/dia) e percepção da qualidade do sono (percepção negativa *vs.* percepção positiva) foram mensuradas por questionário. O estado nutricional foi avaliado pelo índice de massa corporal (baixo peso/peso normal *vs.* sobrepeso/obesidade). A regressão logística binária foi utilizada para analisar a associação entre as variáveis.

**Resultados:**

A prevalência de duração insuficiente e a percepção negativa da qualidade do sono foram de 12,6% (intervalo de confiança de 95% [IC95%] 10,9-14,4) e 21,0% (IC95% 18,9-23,1), respectivamente. Identificou-se tendência linear de aumento na chance de os adolescentes apresentarem duração insuficiente de sono com o aumento do número de fatores negativos (inatividade física, tempo excessivo de tela, excesso de peso corporal) a que eles estavam expostos (*Odds Ratio* [OR]=4,31; IC95% 1,50-12,48).

**Conclusões:**

Adolescentes expostos simultaneamente a baixos níveis de atividade física, tempo excessivo de tela e excesso de peso tiveram menor duração de sono.

## INTRODUCTION

There has been an increasing interest in investigating sleep patterns in adolescents.^1^ ^,^ ^2^ ^,^ ^3^ ^,^ ^4^ This may be due to the fact that biopsychosocial, cognitive and behavioral changes in this phase of life can negatively influence the duration and quality of one’s sleep.^5^ Inadequate sleep can produce health problems, such as poor school performance,^6^ inadequate eating habits,^2^ cardiometabolic risk markers,^7^ obesity and insulin resistance.^8^

The National Sleep Foundation^9^ recommends that adolescents sleep at least eight hours of sleep a day to avoid health problems, however some studies^10^ ^,^ ^11^ have identified that approximately 70% of adolescents have short sleep duration (<8 h/day) and 16.7%^12^ have a negative perception as to the quality of their sleep. In Brazilian adolescents, the prevalence of short sleep duration and the negative perception of sleep quality increased in 10 years from 31.2% in 2001 to 45.9% in 2011.^2^

Low levels of physical activity,^3^ excessive screen time (television, video games and computer)^2^ ^,^ ^4^ and being overweight^13^ are associated with negative perception and not enough sleep in adolescents. However, most of these studies were with older adolescents (14-19 years old),^2^ ^,^ ^14^ ^,^ ^15^ ^,^ ^16^ that have social and behavioral characteristics that are different from younger ones,^17^ in unrepresentative samples. Such studies measured specific sedentary behaviors,^16^ and did not consider simultaneous exposure to physical inactivity, excessive screen time and being overweight.^2^ ^,^ ^3^ ^,^ ^4^ ^,^ ^10^

Analyzes involving the possible associations of simultaneous exposure to these factors can contribute to a better understanding of the possible influences of these factors on the duration and perception of sleep quality in adolescents. Thus, this study determined the prevalence of negative perception of sleep quality and short sleep duration and analyzed whether physical inactivity, excessive screen time and being overweight are associated with these outcomes in adolescents.

## METHOD

Cross-sectional study that used baseline data (2014) from the Longitudinal Study on Sedentary Behavior, Physical Activity, Eating Habits and Adolescent Health (LONCAAFS). LONCAAFS aimed to analyze the interrelationships between level of physical activity, sedentary behaviors, eating habits, quality of life and health indicators of adolescents of both sexes from public schools in the city of João Pessoa (PB), in northeastern Brazil.

To determine the sample size, the following parameters were considered: estimated population of 9,520 students enrolled in the 6th year of elementary school in state and municipal schools; an outcome prevalence of 50%;^18^ a 95% confidence interval (95%CI); a maximum acceptable error of four percentage points; a design effect equal to two; and a 40% increase to compensate for losses and refusals. This resulted in a sample of 1,582 adolescents.

The sampling was made using a single stage conglomerate: systematic selection of 28 public schools, 14 municipal and 14 state schools, proportionally distributed by geographic region of the municipality (north, south, east and west) and number of students in the 6th year of elementary school. In the selected schools, all students were invited to participate in the study.

Data collection took place from February to December 2014 and was performed by trained staff at the school itself and during the adolescent’s study period. The questionnaire was applied through a face-to-face interview, in a room reserved specifically for this purpose.

The sociodemographic variables measured were: sex (male and female); age, in full years, determined by the difference between date of birth and the date of data collection (categorized as: 10-11 and 12-14 years old); and mother’s education, categorized as some elementary school, completed elementary school, and completed high school or higher. Economic class was determined based on criteria from the Brazilian Association of Research Companies (*Associação Brasileira das Empresas de Pesquisa* - ABEP),^19^ grouped into A/B (upper class) and C/D/E (lower middle class) and school period (morning and afternoon).

Physical activity was measured using the Adolescent Physical Activity Questionnaire (*Questionário de Atividade Física de Adolescente* - QAFA)^20^ (reproducibility - intraclass correlation coefficient [ICC]=0.73; p<0.001; validity - Spearman correlation=0.37; p<0.001). The adolescents reported the frequency (days/week) and duration (minutes/day) of their moderate to vigorous intensity physical activities for at least ten minutes, in the week before data collection. The total time of physical activity (minutes/week) was determined by the sum of the products of frequency times the amount of practice time for each activity. Adolescents that exercised less than 300 minutes per week were classified as physically inactive.

To measure sedentary behavior (h/day - ICC=0.69; p<0.01; ≤2 vs. >2 h/day - *Kappa=*0.52), the adolescents reported their time spent on screen activities, such as watching television, playing video games and using the computer, on weekdays and weekends, with the week prior to data collection as the reference period. The weighted average was calculated by adding the average number of hours of screen activities on weekdays and then multiplying it by five, and then by two for weekend days, ultimately dividing the result by seven. Excessive screen time was defined as spending more than two hours a day in these behaviors.^21^

Nutritional status was determined using body mass index (BMI=body mass [kg]/height [m]^2^), by means of measurements of body mass and height. BMI classification was based on World Health Organization (WHO) criteria:^22^ adolescents that are not overweight (underweight+normal weight) and adolescents that are overweight (overweight+obese).

A 24-hour recall was used to measure food consumption. The adolescents reported the food and drinks that they had consumed in the last 24 hours, the preparation method, and the weight and size of the portions. Food consumption data were tabulated using Virtual Nutri Plus software, and total calorie intake values were analyzed using the Food and Nutrition Board of Washington equation.^23^ For the present study, the following food consumption indicators were taken into account: lipid intake values (grams), total saturated fat (g), cholesterol (mg), sodium (mg), and fibers (g). Replication of the 24-hour recall was performed in 30% of the sample to assess intrapersonal variability of the diet and to increase the accuracy of the dietary intake estimate .

To estimate sleep duration per day, the adolescents reported the time they went to sleep and woke up on weekdays (Monday to Friday) and on the weekend (Saturday and Sunday). The average number of hours of sleep per day was calculated by multiplying the number of hours of sleep on weekdays by five and by two for weekend days, and then dividing the result by seven. Short sleep duration was considered to be lasting less than eight hours a day.^9^

Perception of sleep quality was assessed based on the question “In general, how do you assess the quality of your sleep?”, with the following response options: poor, normal, good, very good and excellent. For analysis purposes, this variable was recategorized as negative (bad and normal) and positive (good, very good, excellent) sleep quality. The questions regarding duration (ICC=0.91) and sleep quality (*Kappa*=0.59) obtained satisfactory levels of reproducibility.

The data were entered twice in the EpiData 3.1 program, with automatic checking of the consistency and amplitude of the values. The tool “validate double typing” of this program was used to identify possible typing errors, which were corrected based on the original values of the answers displayed in the questionnaires.

The exclusion criteria adopted were: adolescents who were outside the age group of interest in the study (<10 and ≤14 years of age); those who had a disability that limited the completion of the questionnaire; pregnant teenagers; those who did not answer the questions regarding duration and quality of sleep; those who did not take measurements of body mass and height or physical activity; and those who exhibited sedentary behaviors.

For the descriptive analysis of the data, the mean and standard deviation for quantitative variables and distribution by absolute and relative frequencies for qualitative variables were used. Binary logistic regression analysis was used to assess the crude and adjusted association between the independent variables (physical activity: physically active=0 and physically inactive=1; excessive screen time: no=0 and yes=1; nutritional status: not overweight=0 and overweight=1) and the dependent variables (sleep duration: ≥8 h/day=0 and <8 h/day=1; and sleep quality perception: positive perception=0 and negative perception=1).

Simultaneous exposure to negative factors - being physically inactive, excessive screen time and being overweight - was defined as follows: unexposed, exposed to one, two and three factors, and different combinations of exposure to these factors were also analyzed.

All variables in the crude model were considered for the adjusted analysis. The Forward method was applied for the selection of the variables in the multiple model, and the variables that contributed to the best fit of the model remained (least residual, adjustment of at least 10% in the values of the *Odds Ratio* [OR]). The Hosmer-Lemeshow test was used to assess the fit quality of the model. Interaction analyzes were conducted to assess whether the association between simultaneous exposure to negative factors and the quality and duration of sleep was different between sexes, age groups and school periods.

Potential confounding factors were identified: sex, age, economic class, school period, mother’s education level, and food consumption (values of lipid, total saturated fat, cholesterol, sodium and fiber intake). The analyzes were performed using the Stata 13.0 statistical program, and the level of significance adopted was p≤0.05 for all hypothesis tests.

The LONCAAFS study was approved by the Human Research Ethics Committee of the Health Sciences Center of the Universidade Federal da Paraíba (Protocol nº 0240/13). All adolescents who participated in the study were authorized to do so by their father/mother/guardian.

## RESULTS

A total of 2,767 adolescents were invited to participate in this study. Of these, 830 (30.0%) did not return the informed consent form, 372 did not agree to participate in the study (13.4%), and 133 were not found in at least three visits made by the data collection team. The final sample of this study, thus included 1,432 adolescents. No significant differences were identified (p≤0.05) between the adolescents included and those excluded from the analyzes. The calculation of statistical power *a posteriori* (α=0.05 and ß=0.20) indicated that this sample allowed for the detection of significant OR values that were equal to or greater than 1.9, with the prevalence of the outcome in those not exposed, varying from 12.6 to 20.6%.

Most of the adolescents were female (53.1%), from low- and middle-income classes (65.8%) and were exposed to excessive screen time (59.6%). Approximately 35% of adolescents were classified as physically inactive and 32.5% were overweight (Table 1). The prevalence of short sleep duration and negative perception of sleep quality were 12.6 (95% confidence interval - 95%CI 10.9-14.4) and 21.0% (95%CI 18.9-23.1), respectively, being higher in the youngest (p<0.001) (data not shown in the table). It was observed that 44.8% of the adolescents had a negative factor, 33.4% presented two negative factors and 6.6% presented three negative factors (data not included in the table). As for the simultaneous exposure of negative factors, it was seen that 44.8% of the adolescents indicated a negative factor, 33.4% indicated two negative factors and 6.6% indicated three negative factors (data not shown in the table).

**Table 1:** Sociodemographic characteristics, physical activity, screen time and nutritional status of adolescents in João Pessoa, Paraíba, 2014*.

	n	%
Sex		
Male	675	46.9
Female	763	53.1
Age group		
10 to 11	816	56.7
12 to 14	622	43.3
Economic class		
A/B	428	34.2
C/D/E	823	65.8
Mother’s level of education		
Some elementary school	484	40.6
Completed elementary school	339	28.5
Completed high school or higher	368	30.9
School period		
Morning	638	44.3
Afternoon	800	55.7
Screen time		
≤2 h/day	581	40.4
>2 h/day	857	59.6
Television		
≤2 h/day	940	65.0
>2 h/day	498	35.0
Computer/video games		
≤2 h/day	1.129	89.6
>2 h/day	149	10.4
Level of physical activity		
Physically active	960	66.8
Physically inactive	478	33.2
Nutritional status		
Not overweight	963	67.5
Overweight	463	32.5

*The sum does not correspond to the total number of cases in the sample due to non-existent values (*missing*).

Excessive screen time (OR=1.96; 95%CI 1.18-2.80) was significantly associated with short sleep duration. The combination pattern for excessive screen time was associated with all possible combinations of short sleep duration (Table 2). There were no significant interactions between sex, age and school period with quality and duration of sleep (p>0.05).

**Table 2:** Crude and adjusted logistic regression analysis for association between physical activity level, screen time and nutritional status with short sleep duration and negative perception of sleep quality in adolescents, João Pessoa, Paraíba, 2014.

Variables				Short sleep duration (n=181)			Negative perception of sleep quality (n=300)		
				n (%)	Crude	Adjusted*	n (%)	Crude	Adjusted
Number of factors	PI	EST	O		OR (95%CI)	OR (95%CI)		OR (95%CI)	OR (95%CI)
0	-	-	-	25 (9.7%)	1	1	52 (20.2%)	1	1
1	+	-	-	12 (8.0%)	1.34 (0.97-1.83)	0.88 (0.60-1.29)	25 (16.7%)	1.25 (0.96-1.63)	0.83 (0.57-1.23)
1	-	+	-	52 (13.8%)	1.43 (1.03-1.99)	1.96 (1.16-3.30)	89 (23.6%)	1.86 (0.65-1.13)	0.93 (0.65-1.33)
1	-	-	+	18 (14.8%)	1.33 (0.96-1.85)	1.33 (0.82-2.15)	18 (16.5%)	0.90 (0.68-1.19)	1.17 (0.82-1.66)
2	+	+	-	20 (11.5%)	1.15 (0.76-1.85)	2.18 (1.03-4.67)	40 (23.0%)	1.07 (0.72-1.58)	0.96 (0.55-1.67)
2	-	+	+	32 (16.0%)	1.90 (1.19-3.00)	2.68 (1.31-5.47)	46 (23.0%)	1.12 (0.76-1.63)	1.09 (0.66-1.79)
2	+	-	+	4 (7.1%)	1.04 (0.61-1.78)	1.29 (0.58-2.88)	12 (21.4%)	0.76 (0.48-1.20)	0.76 (0.41-1.40)
3	+	+	+	25 (9.7%)	1.38 (1.17-1.63)	4.18 (1.39-12.5)	32 (20.2%)	0.69 (0.39-1.22)	0.75 (0.32-1.76)

+Presence of the factor; -Absence of the factor; PI: physical inactivity; EST: excessive screen time; O: overweight; OR: *Odds Ratio*; 95%CI: 95% confidence interval; *analyses adjusted for sex, age, class period, economic class, parental education level, food consumption, screen time, nutritional status, physical activity level and other variables in the model.

**Figure 1:**
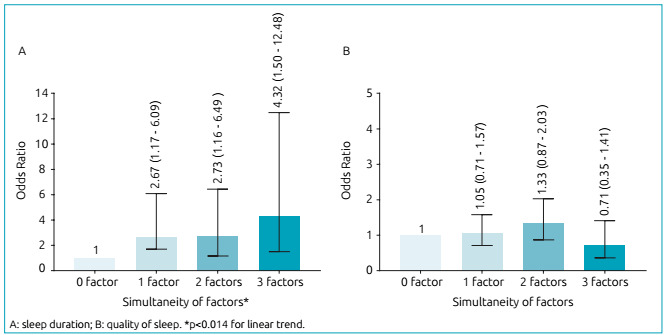
Simultaneous exposure to negative factors for short sleep duration and negative perception of sleep quality in adolescents, João Pessoa, Paraíba, 2014.

There was a linear tendency to increase the chance (OR=4.31; 95%CI 1.50-12.48) of adolescents having short sleep duration as they were simultaneously exposed to physical inactivity, excessive screen time and excess body weight (Figure 1). The result of the Hosmer-Lemeshow test (chi-square=6.32; p=0.412) demonstrated that the final model of analysis adjusted well to the data.

## DISCUSSION

The prevalence of negative perception on sleep quality and short sleep duration was high, and adolescents who mentioned excessive exposure to screen time were more likely to not have enough sleep. Another important finding of this study was that it identified a linear tendency to increase the chance of adolescents not having enough sleep as they were simultaneously exposed to physical inactivity, excessive screen time and being overweight.

The prevalence of short sleep duration observed here was lower than that of other international studies (United States - 68.7%,^10^ Canada - 70.0%)^21^ and Brazilian studies in the South (Santa Catarina - 54.0%),^1^ Southeast (São Paulo - 39.0%),^24^ Northeast (Caruaru - 77.1%)^15^ and Northern Brazil (Amazonas - 51.2%).^14^ The proportion of negative perception of sleep quality in the present study was also lower than that of foreign countries^12^ ^,^ ^25^ and other Brazilian regions.^1^ ^,^ ^15^

A probable explanation for this variability in the results is the fact that adolescent sleep is influenced by socioeconomic, cultural and behavioral differences, which seem to be decisive in altering the duration and quality of sleep in adolescents.^2^ ^,^ ^14^ Differences in sample characteristics should also be considered, since, in this study, about 60% of adolescents were aged up to 11 years old. This was different from the other studies in which the age range age ranged from 14 to 19 years old. ^2^ ^,^ ^10^ ^,^ ^14^ ^,^ ^15^ ^,^ ^26^ Older teenagers have more freedom to manage their time with friends, participate in leisure activities until late hours, and socialize more frequently and for longer on electronic devices,^17^ which can contribute to inadequate sleep.

The duration of nighttime sleep among adolescents decreased by an average of two hours a day between 1910 and 2009.^27^ This has been associated with several reasons, such as changes in adolescent sleep patterns, increased school and activity obligations, and high exposure to screen time.^3^ This is a worrying phenomenon from the point of view of public health, given that not enough daily sleep contributes to a lower quality of sleep,^15^ an increase in daytime sleepiness,^24^ unhealthy eating habits,^2^ and low school performance.^6^

It was found that the likelihood of an adolescent not having enough sleep increased linearly as he or she was simultaneously exposed to low levels of physical activity, excessive screen time, and being overweight. These results corroborate those observed by Kim et al.,^4^ who indicated that adolescents who were physically more active and less exposed to sedentary screen behaviors were more likely to not get enough sleep. Such findings reinforce the hypothesis that the greater the number of risk factors to which adolescents are exposed, thegreater the chance of them having their health levels and/or their indicators compromised, including sleep.^4^ ^,^ ^7^ Therefore, health promotion interventions, including improving the quantity and quality of sleep, must also perform simultaneous actions to increase levels of physical activity and reduce body weight and excessive screen time.

Excessive screen time (television/computer/video games) was significantly associated with short sleep duration. This result can be considered the main finding of this study. It reinforces the available evidence on the harmful effects of sedentary behavior on sleep in adolescents.^2^ ^,^ ^10^ ^,^ ^15^ ^,^ ^28^ ^,^ ^29^ A systematic revision observed that in 90% of the analyzed studies, greater exposure to screen time was linked to short sleep duration.^3^

Different mechanisms may explain the deleterious effects of time spent in sedentary behavior with regard to sleep in adolescents. The use of devices tends to extend for long periods of the night, reducing the amount of time sleeping.^29^ This can cause changes in sleep architecture, leading adolescents to have a negative perception of the quality of their sleep. Exposure to bright screens close to bedtime can affect the sleep cycle, by means of nocturnal salivary suppression of melatonin, decreasing sleep duration.^28^ As such, the American Academy of Pediatrics^30^ recommends that children and adolescents limit their exposure time to television, computers, and videogames to a maximum of two hours a day. Therefore, it is important to monitor young people’s exposure to sedentary screen behaviors, especially at night.

The main limitation of the present study was that it did not include adolescents from the private school system, limiting the ability to generalize its findings. It is widely documented that age and socioeconomic status can influence the quality and duration of adolescents’ sleep.^1^ Because this is a cross-sectional study, it was not possible to establish a causal relationship between level of physical activity, excessive screen time, being overweight and excess quantity and quality of sleep.

On the other hand, this study is one of the first to analyze the simultaneous relationship of insufficient levels of physical activity, excessive screen time and being overweight in the duration and quality of sleep in adolescents. It was carried out in a representative sample of students from the 6th grade of a public elementary school and it used previously tested instruments with acceptable levels of reproducibility and validity, applied by trained people who followed a uniform collection protocol.

It was concluded that the prevalence of adolescents with a negative perception of quality and short sleep duration was relatively high. Furthermore, simultaneous exposure to excessive screen time, low levels of physical activity and being overweight increased adolescents’ chances of not getting enough sleep.
